# Targeting the PI3K/Akt pathway for the treatment of ulcerative colitis: integrative regulatory features of traditional Chinese medicine

**DOI:** 10.3389/fphar.2025.1620138

**Published:** 2025-10-09

**Authors:** Siying Niu, Yang Zhang

**Affiliations:** ^1^ First Clinical Medical College, Heilongjiang University of Chinese Medicine, Harbin, China; ^2^ Department of Gastroenterology, First Affiliated Hospital, Heilongjiang University of Chinese Medicine, Harbin, China

**Keywords:** ulcerative colitis, traditional Chinese medicine, PI3K/Akt signaling pathway, inflammatory response, oxidative stress, autophagy and apoptosis, intestinal barrier, gut microbiota

## Abstract

The development of ulcerative colitis (UC), a highly prevalent inflammatory bowel disease worldwide, is closely related to the key regulatory role of the PI3K/Akt signaling pathway. Although the use of Western drugs is the standard clinical treatment, the problems of drug resistance, side effects, and efficacy limitations are prominent. In contrast, Traditional Chinese Medicine (TCM), with the advantages of multi-target regulation and low toxicity and side effects, demonstrates the potential for and offers highly effective treatment. This paper systematically analyzes the mechanism of the PI3K/Akt pathway in UC. It focuses on how TCM metabolites, extracts, and formulas regulate inflammatory responses, oxidative stress, autophagy and apoptosis, intestinal barrier function, and gut microbiota homeostasis by targeting the pathway, which together can achieve the goal of alleviating the symptoms of UC. Our goal is to provide new insights into the prevention and treatment of UC and to contribute to the standardization of TCM.

## 1 Background

Ulcerative colitis (UC) is a chronic inflammatory bowel disease involving the mucosa of the colon and rectum. Typical symptoms include persistent diarrhea, mucopurulent and bloody stools, and abdominal pain. The incidence of UC is increasing year by year, reaching about 5 million cases worldwide in 2023 (Le Berre et al., 2023), mainly in Asia, South America, and southern North America (Zhang Y. et al., 2024). The prevalence of UC in China increased from 0.51/100,000 to 8.95/100,000 between 2000 and 2016 (Xu L. et al., 2023; Wan et al., 2024), with a significant increase in the urban youth population, which is expected to continue to climb with social development. Current evidence indicates that the pathogenesis involves a multifactorial interaction of genetics, infection, dysbiosis, immune abnormalities, and diet.

Western medicine mainly uses 5-aminosalicylic acid (5-ASA), glucocorticoids, immunosuppressants, and biologics, which can relieve symptoms but have significant limitations. 5-ASA drugs (e.g., mesalamine) have limited efficacy in mild-to-moderate UC and are susceptible to drug resistance. Long-term use of immunosuppressive agents (e.g., azathioprine) can lead to immunosuppression and increase the risk of infection (Olivera et al., 2020) and malignancy. Biological agents (e.g., anti-TNF-α antibody) are effective for severe UC, but high cost and side effects such as allergy, immunity, and tolerance limit the application (Luo et al., 2022). The efficiency of existing drug therapy is only 30%–60% (Gros and Kaplan, 2023), and new strategies are urgently needed.

Globally, in addition to TCM, traditional medicine systems such as the Indian Ayurveda (Javali and Thirumurugan, 2024) and Traditional Mexican Medicine (Soto et al., 2023) have also accumulated abundant experience in the treatment of chronic inflammatory diseases such as UC, especially in the application of botanicals, which show multi-targeted and low-toxicity therapeutic characteristics, and have a lot of similarities with TCM. Despite the differences in mechanisms and theoretical systems, TCM is relatively more systematic and reproducible, particularly in the standardization of preparations, extraction processes, and compounding theories, which provides a solid foundation for its modernization (Xue et al., 2022; Yagüe et al., 2022). TCM is a medical system based on unique Chinese cultural theories and practices, with more than 2000 years of effective treatment history. Its multi-component synergistic and multi-pathway regulation treatment model has significant advantages in UC immunomodulation, mucosal repair, and flora regulation (Kou et al., 2020; Feng et al., 2022; Zhang X. et al., 2022; Zhang et al., 2024d; Zhang et al., 2024b). In this paper, we systematically analyze the regulatory role of the PI3K/Akt pathway in UC, with a particular focus on the diverse active metabolites, extracts, and formulas derived from TCM that modulate this pathway, thereby providing a theoretical basis for therapeutic strategies in UC.

## 2 Role of the PI3K/Akt signaling pathway in UC

**FIGURE 1:**
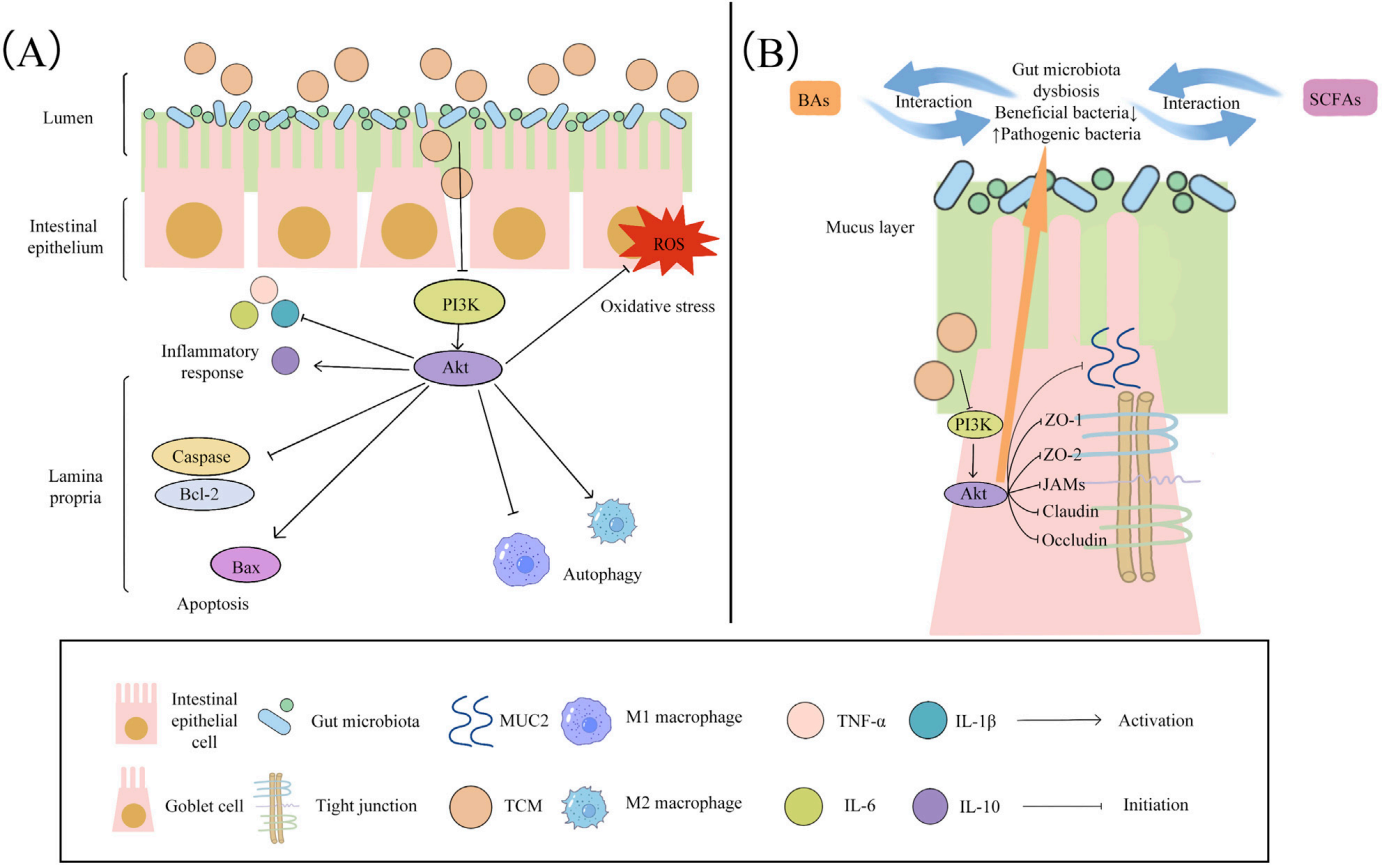
Mechanisms of TCM regulating the PI3K/Akt signaling pathway for the treatment of UC: **(A)** TCM modulates the PI3K/Akt signaling pathway to reduce pro-inflammatory cytokines such as TNF-α, IL-1β, and IL-6 while increasing levels of IL-10; inhibits oxidative stress; inhibits M1 macrophage polarization and promotes M2 macrophage polarization to regulate autophagy; and decreases the levels of Bcl-2 and Caspase while increasing the level of Bax to regulate apoptosis. **(B)** TCM activates PI3K/Akt to restore the intestinal barrier by up-regulating tight junction proteins (ZO-1, ZO-2, JAMs, Claudin, and Occludin) and mucin MUC2, and rebalances the gut microbiota through the BAs–microbiota axis and the SCFAs–microbiota axis.

The specific etiology and pathogenesis of UC have not been fully clarified. Among the many signaling pathways associated with UC, abnormal activation of the PI3K/Akt signaling pathway may be one of the important mechanisms in the pathogenesis of UC, which has attracted attention for its multiple roles in regulating inflammatory responses, reducing oxidative stress, modulating apoptosis and autophagy, repairing the intestinal mucosal barrier, and maintaining the balance of gut microbiota (Figure 1).

Inflammatory response is the central pathologic process in the pathogenesis of UC. In UC, the balance between pro-inflammatory and anti-inflammatory cytokines is disrupted (Tatiya-aphiradee et al., 2019). Major pro-inflammatory cytokines such as TNF-α, IL-1β, and IL-6, which activate the immune system, induce more immune cells to accumulate at the site of inflammation and increase the inflammatory response in the gut. At the same time, anti-inflammatory cytokines such as IL-10 are diminished, allowing inflammation to persist. Excessive inflammatory responses lead to intestinal tissue damage. Cytokines not only promote the inflammatory response by activating immune cells but also increase the permeability of intestinal epithelial cells, which impairs the intestinal barrier and further exacerbates the local immune response. This vicious cycle of local immune response leads to intestinal mucosal injury, ulcer formation, and bleeding. These pathological changes eventually result in the clinical symptoms of UC, such as diarrhea, abdominal pain, and bloody stools (Yao et al., 2019). Studies have shown that abnormal activation of the PI3K/Akt signaling pathway triggers NF-κB activity through phosphorylation of IκB. This disturbance disrupts cytokine homeostasis, driving the overproduction of pro-inflammatory mediators such as TNF-α, IL-1β, and IL-6, while reducing IL-10, an important anti-inflammatory cytokine. Blocking this signaling pathway can attenuate the inflammatory response and alleviate the symptoms of UC (Antoniv and Ivashkiv, 2011; Huang et al., 2011).

Abnormal activation of oxidative stress has been shown to be a pathological mechanism in the development of UC (Tian et al., 2017). This disorder arises from an imbalance between the generation of Reactive Oxygen Species (ROS) and the body’s antioxidant defense system. As ROS accumulate, they induce a series of damaging processes, including lipid peroxidation, protein denaturation, and DNA injury. In the course of UC, this oxidative/antioxidative imbalance mainly involves the following mechanisms: infiltration of intrinsic immune cells neutrophils and macrophages into the inflamed intestine, which releases large amounts of ROS through the NADPH oxidase (NOX)-dependent pathway; mitochondrial dysfunction in damaged intestinal epithelial cells leading to abnormalities in the electron transport chain, which generates excessive superoxide anion production; and the production of local antioxidants in the intestine, such as glutathione (GSH), superoxide dismutase (SOD), and other levels are significantly reduced, weakening the body’s ability to scavenge free radicals (Muro et al., 2024). Inhibition of the PI3K/Akt signaling pathway, increase of SOD and glutathione peroxidase (GSH-XP) activities, and reduction of malondialdehyde (MDA) content have important protective roles in the oxidative stress response in UC (Yan et al., 2020). Studies have shown that Hy-Exos can significantly elevate the expression levels of catalases (CAT) and reduce GSH in colon tissues by delivering miR-214-3p to specifically regulate the PI3K/Akt/mTOR signaling axis and at the same time inhibiting the abnormal generation of ROS, thus effectively reestablishing the oxidative/antioxidative balance of the intestinal tract (Li N. et al., 2024).

Macrophage autophagy affects intestinal homeostasis by regulating inflammatory responses. Studies have shown that AMPK activation promotes macrophage autophagy, suppresses inflammation, and alleviates experimental colitis (Banskota et al., 2023). When ATG16L1 is absent, macrophages lose the ability to induce regulatory T cells, leading to elevated levels of pro-inflammatory cytokines and, consequently, increased intestinal inflammation (Chu et al., 2016). Inhibition of autophagy drives macrophage polarization toward the pro-inflammatory property M1 phenotype, which disrupts the epithelial barrier by disassembling tight junction proteins, triggering the accumulation of apoptotic cells and an excessive inflammatory response. In contrast, the M2 phenotype, which has anti-inflammatory properties, promotes tissue repair by clearing up inflammation (Zhang M. et al., 2023). Thus, defective or inhibited macrophage autophagy exacerbates intestinal inflammation. The PI3K/Akt pathway plays a key role in UC by regulating mTORC activity; when the pathway is hyper-activated, it suppresses autophagy and thereby intensifies the inflammatory response (Zhang et al., 2024a). It has been confirmed that vildagliptin activates autophagy and promotes tissue repair by inhibiting the PI3K/Akt/mTOR pathway (Awad, 2024). In addition, the probiotic *Christensenellaceae minuta* (*C. minuta*) inhibits the PI3K-Akt pathway by upregulating IGF-1 expression, promotes macrophage polarization toward M2, and reduces pro-inflammatory factor production (Yao et al., 2024).

Abnormal regulation of apoptosis, a key metabolic process for maintaining intestinal homeostasis, is closely associated with intestinal mucosal barrier damage in UC. Excessive apoptosis of intestinal epithelial cells and delayed apoptosis of inflammatory cells together lead to intestinal barrier dysfunction (Wan et al., 2022). Endoplasmic reticulum stress induces apoptosis through a dual mechanism: on the one hand, by activating caspase-12 and caspase-9 through disruption of Ca^2+^ homeostasis; on the other hand, by upregulating the expression of the pro-apoptotic proteins, the Bcl-2 family of proteins, which together induce apoptosis (Xu, 2005). The PI3K/Akt pathway, as a core anti-apoptotic regulatory axis, plays a key role in maintaining the integrity of the intestinal barrier by regulating effector molecules, such as Bcl-2 family proteins and caspase-3 (Jalil et al., 2023). In the present study, we found that *Citrus unshiu* peel (Chen pi) water extract (CUP) upregulated the expression of anti-apoptotic proteins Bcl-2 and Survivin by significantly inhibiting the expression of pro-apoptotic proteins Bax and caspase-3 in colonic tissues. Thus, CUP regulates the expression of apoptosis-related proteins by regulating the PI3K/Akt signaling pathway, which in turn exerts anti-inflammatory and protective effects on the intestinal mucosa (Lee et al., 2022).

The intestinal mucosa is an important defense system in the intestinal tract, which can effectively isolate harmful substances in the intestinal tract to maintain the barrier function and intestinal homeostasis, including mechanical barrier, chemical barrier, biological barrier, and immune barrier. Among them, mucin 2 (MUC2) secreted by goblet cells constitutes the mucus layer covering the intestinal epithelium, which not only prevents mechanical injuries and harmful substances at the physical level but also neutralizes or defends against pathogens and toxins in the intestinal lumen through the function of the chemical barrier, thus playing a crucial role in the intestinal barrier. The mechanical barrier consists of the connections between the colonic epidermal cells and the epidermal cells, including tight junctions (TJ), gap junctions, and adhesion junctions (AJ). TJ consists of a variety of proteins, among which the transmembrane protein Occludin and the cytoplasmic protein Zonula occludens-1 (ZO-1) are key proteins for maintaining the structure of the TJ and the function of the intestinal epithelial barrier (Xu Q. et al., 2023; Zuo et al., 2020; Fasano, 2011). In UC, tight junction proteins and mucins are pathologically altered (Cantero-Recasens et al., 2022; Hu et al., 2021; He et al., 2020), and the PI3K/Akt signaling pathway can improve intestinal barrier function by regulating the expression of these proteins. Experiments showed that in the LPS-induced Caco-2 cell barrier injury model, Ferulic Acid (Fu et al., 2022) restored the expression of Occludin and ZO-1, thereby maintaining intestinal barrier integrity. This effect was achieved through the upregulation of miR-200c-3p, which in turn suppressed PTEN expression and ultimately enhanced PI3K/Akt signaling.

Patients with UC are often characterized by an imbalance of the gut microbiota. This is mainly reflected in reduced microbial diversity, a decline in beneficial bacteria such as *Firmicutes*, *Akkermansia*, and *Bifidobacterium*, and an overgrowth of pathogenic bacteria including *Bacteroidetes*, *Escherichia coli*, and *Clostridium perfringens*. This dysbiosis leads to reduced synthesis of short-chain fatty acids (SCFAs), which promotes TNF-α secretion through activation of the NF-κB pathway while inhibiting Treg cell differentiation, ultimately exacerbating intestinal epithelial barrier damage (Fu et al., 2022; Hu et al., 2019; Shen et al., 2018). More and more studies have found that PI3K/Akt can directly or indirectly regulate the gut microbiota to alleviate UC. Resveratrol (RSV) improves the structure of the gut microbiota in UC mice by directly inhibiting the activation of the PI3K/Akt signaling pathway and by increasing the number of beneficial bacteria, such as *Akkermansia* (Zhu et al., 2021). The PI3K/Akt signaling pathway indirectly regulates gut microbiota balance by regulating bile acids (BAs) and SCFAs. BAs metabolism interacts bi-directionally with the gut microbiota to form the bile acid–gut microbiota axis lowercase letters. Bile salt hydrolase (BSH) and bile acid-inducible enzyme (BAI) are key bacterial enzyme systems that regulate bile acid metabolism in bacteria mainly found in the *Firmicutes*. BAs are converted to secondary bile acids (e.g., deoxycholic acid, lithocholic acid) by BSH and BAI. BAs, in turn, affect flora growth and metabolism through receptors such as Farnesoid X receptor (FXR) and Takeda G-protein receptor 5 (TGR5) (Yang et al., 2021). SCFAs (e.g., acetic acid, propionic acid, butyric acid) are the main products of gut microbiota to metabolize dietary fiber, and these metabolites not only regulate flora composition but also enhance intestinal barrier function and immunomodulation. SCFAs and gut microbiota regulate each other. SCFAs can promote the growth of anaerobic bacteria and influence the intestinal environment by lowering the pH value, inhibiting pathogenic bacteria, and lowering the redox potential. In addition, probiotics such as *Lactobacillus* and *Bifidobacterium* produce acetic acid and lactic acid during the proliferation process, and these metabolites can be utilized as substrates by other bacteria (e.g., *Faecalibacterium prausnitzii*) to produce butyric acid, thus constructing a micro-ecosystem of mutual nourishment and functional synergy (Yap et al., 2021; Yang S. et al., 2022; Ambat et al., 2024). Herba Origani (Niu zhi) Extract Pulvis (HOEP) modulates the PI Pathway, affects BAs metabolism and SCFAs metabolism, and restores alpha diversity of the intestinal microbiota and immune system homeostasis (Yu et al., 2023).

## 3 Recognition and treatment of UC in Chinese medicine

UC belongs to the category of “dysentery” in Chinese medicine. TCM has a long history of treating UC. According to the theory of etiology and pathogenesis of Chinese medicine, dampness, and heat play a dominant role. External influences, dietary irregularities, or emotional disorders cause dampness and turbidity to obstruct and heat to injure the intestinal tract of UC for a long period, resulting in damage to the spleen and kidneys, qi and yang deficiencies, blood and qi stagnation, and internal ulceration into ulcers, which are mixed and mixed with the lower part of the body. The disease is related to the accumulation of dampness and heat, blood stasis, insufficient spleen qi, insufficient kidney yang, and the mixture of cold and heat, so the treatment is to clear away heat and dry dampness, activate blood circulation and remove blood stasis, warm the spleen and kidneys, and harmonize cold and heat as the conventional method.

Based on the above TCM theory, several classical formulas, such as Gegen Qinlian Decoction (Zhao et al., 2021; Wang et al., 2023), Fructus mume pills (Wumei Wan, FMPs) (Yan et al., 2022; Chen et al., 2023), Pulsatilla Decoction (Baitouweng Tang) (Yu et al., 2021; Xie et al., 2024), and Huangqin Decoction (Mo et al., 2022; Wu et al., 2022), are derived from ancient Chinese medical classics and have shown promising clinical results in UC treatment. Meanwhile, the major botanical drugs in these formulas, including *Pueraria montana var*. *lobata* (Willd.) Maesen and S.M.Almeida ex Sanjappa and Predeep (Gegen) (Ga et al., 2024), *Paeonia lactiflora* Pall. (Baishao) (Zheng et al., 2018; Hu et al., 2024), *Scutellaria baicalensis* Georgi (Huangqin) (Li Y. et al., 2022; Chong et al., 2025), *Coptis chinensis* Franch. (Huanglian) (Wang et al., 2021; Yang Y. et al., 2022), *Rheum palmatum* L. (Dahuang) (Luo et al., 2022; Zhang Y. et al., 2023), and *Sophora flavescens* Aiton (Kushen) (Li Z. et al., 2022; Sun et al., 2024), have been rigorously studied. These botanical drugs consistently showed powerful anti-UC properties. Further studies have shown that the therapeutic effects of these botanical drugs on UC are mainly attributed to their active metabolites, such as *Pueraria lobata* polysaccharide, Paeoniflorin, Baicalin, Berberine, Rhein, Oxymatrine, and more. Through extensive *in vitro* and *in vivo* experiments, it has been demonstrated that botanical drugs and their active metabolites treat UC mainly by modulating the PI3K/Akt signaling pathway, thus supporting the clinical application of classical TCM formulas.

## 4 TCM metabolites and their role in modulating the PI3K/Akt pathway in UC treatment

As one of the treasures of traditional Chinese culture, TCM stands out with its unique diagnostic and therapeutic methods and proven clinical efficacy. TCM treatment of UC has advantages in stabilizing the disease, improving symptoms, preventing recurrence, and improving patients’ quality of life (Che et al., 2022; Zheng et al., 2022; Liu et al., 2024; Wang et al., 2024). A growing number of studies have confirmed the potential of botanical drugs in the treatment of UC. These metabolites may act by modulating the balance of pro-inflammatory and anti-inflammatory factors, decreasing the level of oxidative stress, regulating autophagy and modulation, repairing the intestinal mucosal barrier, and modulating intestinal microbial homeostasis. Notably, many of these effects are mediated through modulation of the PI3K/Akt signaling pathway, making TCM a promising avenue in the field of UC treatment (Supplementary Table 1).

### 4.1 Modulation of the inflammatory response

TCM metabolites have shown significant potential in regulating the inflammatory response in UC by modulating the PI3K-Akt signaling pathway. Paeoniflorin (PF) is a natural metabolite extracted from *Pueraria montana var*. *lobata* (Willd.) Maesen and S.M.Almeida ex Sanjappa and Predeep. Studies have shown that PF can inhibit the release of the inflammatory factors TNF-α and IL-1β by regulating the PI3K-Akt-mTOR signaling pathway and increasing the phosphorylation level of S6K1. It can also upregulate the molecular markers of intestinal stem cells (ISCs), including Lgr5, Sox9, and Ascl2, promoting the renewal and differentiation of ISCs, improving the growth of colon organoids and the regenerative repair capacity of intestinal epithelial cells, and accelerating intestinal mucosal healing (Ma et al., 2023). The network pharmacological analysis showed that dihydrophenanthrene and phenanthrene are the main UC-related metabolites in *Bletilla striata* (Thunb.) Rchb.f. (Baiji). In the DSS-induced UC mouse model, it can improve the disease activity index (DAI) and body weight, prevent colon shortening, and improve colon pathological damage. These improvements are associated with the inhibition of the EGFR/PI3K/Akt signaling pathway, which can reduce the levels of the inflammatory cytokines TNF-α and IL-6 and alleviate the symptoms of UC in mice (Gong et al., 2023). Bisdemethoxycurcumin (BDMC) is a polyphenolic metabolite derived from *Curcuma longa* L. (Jianghuang). It was shown that BDMC significantly reduced the levels of pro-inflammatory cytokines IL-6, IL-1β, TNF-α, and MCP-1 in LPS-induced RAW264.7 cells and that this anti-inflammatory effect is associated with inhibition of the activation of the PI3K/Akt signaling pathway (Wu et al., 2023). Mogroside V (MGV) from *Siraitia grosvenorii* (Swingle) A.M.Lu and Z.Y.Zhang (Luohanguo) exerts an anti-inflammatory effect in the treatment of UC by modulating the AMPK-PI3K/Akt/mTOR signaling pathway. Specifically, MGV enhances the phosphorylation level of AMPK and inhibits the phosphorylation of PI3K and Akt proteins, thereby inhibiting the downstream mTOR signaling pathway. This mechanism of action blocks the expression of pro-inflammatory cytokines such as TNF-α, IL-6, COX-2, AP-1/HO-1, and iNOS, thereby reducing the inflammatory response and tissue damage (Zhou et al., 2021).

### 4.2 Reduction of oxidative stress

Active metabolites from TCM show significant potential in regulating the level of oxidative stress in UC, mainly through their interaction with the PI3K/Akt signaling pathway. Dehydroevodiamine (DHE) is an active alkaloid isolated from *Tetradium ruticarpum* (A.Juss.) T.G.Hartley (Wuzhuyu). DHE reduces the DSS-induced levels of MDA in the serum and colonic tissues of UC rats by inhibiting the activation of the PI3K/Akt/NF-κB signaling pathway. Furthermore, it increased the levels of HO-1 and SOD, reducing the levels of inflammation and oxidative stress in UC rats (Ma et al., 2024). Glycitein in *Codonopsis pilosula* (Franch.) Nannf. (Dangshen) aqueous extract (DS) was identified as a core active metabolite and was directly related to the PI3K/Akt signaling pathway. It was found that high doses of DS had a significant protective effect on the inflammatory response in rats with UC, and DS treatment significantly increased GSH and SOD levels and decreased MPO and MDA levels in rats with UC. In addition, DS was able to significantly restore the activity of ATPase, which could effectively regulate metabolic disorders and enhance antioxidant capacity to ameliorate UC (Li F. et al., 2024). Patchouli essential oil (PEO) from *Pogostemon cablin* (Blanco) Benth. (Guanghuoxiang) has been shown to have therapeutic effects on UC. PEO significantly inhibited the activation of the PI3K-Akt pathway and significantly reduced the levels of TNF-α and IL-1β in the colonic tissues, which attenuated inflammatory responses. The metabolite increased the activity of the antioxidant enzyme GSH and decreased the activity of MPO, suggesting an antioxidant effect. Activation of the PI3K-Akt pathway enhances the activity of Gli-1, a key metabolite of the Hedgehog pathway, suggesting an interaction between the two in the inflammatory response. PEO was able to indirectly modulate the Hedgehog pathway by inhibiting the activation of the PI3K-Akt pathway and further reducing inflammation in UC (Huang et al., 2025).

### 4.3 Regulation of autophagy and apoptosis

By interacting with the PI3K/Akt signaling pathway, the herbal monomers and their metabolites showed significant efficacy in regulating autophagy and apoptosis in UC. The *Sanguisorba officinalis* ethyl acetate fraction (SOEA) of *Sanguisorba officinalis* L. (Diyu) exerts its therapeutic activity through the PI3K-Akt/NF-κB/STAT3 pathway, decreasing the proportion of fibroblasts and macrophages, but increasing the proportion of granulocytes and T cells to varying degrees, while improving clinical symptoms, immune response, and intestinal mucosal barrier in UC (Li et al., 2023). Platycodin D (PLD), a natural product found in *Platycodon grandiflorum* (Jacq.) A.DC. (Jiegeng), has been shown to be an AMPK activator. PLD is involved in the polarization transformation of macrophages by promoting the activation of the PI3K/Akt signaling pathway and inhibiting the activation of the NF-κB signaling pathway, converting M1-type macrophages to M2-type macrophages (Guo et al., 2021). Oxymatrine (OMT) is a major active metabolite extracted from *Sophora flavescens* Aiton. Studies have shown that OMT inhibits the PI3K/Akt pathway, activates its downstream sheared forms of cleaved-caspase3 and cleaved-caspase9, and decreases the expression of Bcl-2 and Bad, inducing apoptosis of inflammatory cells in the colon tissue (Chen et al., 2017). Baicalin (BI) is an active metabolite of *Scutellaria baicalensis* Georgi that prevents excessive apoptosis and promotes repair of mucosal cells by inhibiting PI3K/Akt signaling, elevating the level of Bcl-2 and inhibiting inflammatory response and apoptosis in HT-29 cells (Wang et al., 2022a).

### 4.4 Repair of the intestinal barrier

*Cimicifuga heracleifolia* Kom. (Shengma) has been shown to significantly elevate tight junction proteins Claudin-1, Occludin, and ZO-1, as well as the MUC2, through inhibition of PI3K/Akt/NF-κB signaling pathway to enhance the intestinal mucosal barrier to reduce pathogen invasion (Wu et al., 2025). Berberine (BBR) is the main active metabolite of *Coptis chinensis* Franch. It effectively inhibits the PI3K/Akt/mTOR signaling pathway by targeting IRGM1, restores the expression of intestinal tight junction proteins ZO-1, Occludin, and Claudin-1 expression, protects intestinal barrier function, and reduces serum levels of D-lactate (D-LA) and diamine oxidase (DAO), thereby improving intestinal permeability (Meng et al., 2024). Astragaloside IV is an active flavonoid metabolite extracted from *Astragalus membranaceus* Bunge (Huangqi). It enhances the integrity of the intestinal barrier by inhibiting the phosphorylation levels of PI3K and Akt and promoting the expression of tight junction proteins such as ZO-1, Occludin, Claudin-5, Claudin-7, and Villin (Zhang et al., 2024c). Aloin A is one of the main active metabolites of *Aloe vera* (L.) Burm.f. (Luhui) extracts. In DSS-induced UC rats, it can significantly increase the synthesis and secretion of colon mucus proteins such as MUC2 and MUC5AC in LS174T cells by activating the expression of key proteins in the PI3K/Akt signaling pathway (p-PI3K and p-Akt), thereby thickening the colon mucus layer and improving mucus barrier function (Shi et al., 2021).

### 4.5 Regulation of gut microbiota balance

RSV is an extract of *Smilax glabra* Roxb. (Tufuling). It improves the gut microbiota structure of UC mice by inhibiting the activation of the PI3K/Akt signaling pathway and increasing the number of beneficial bacteria, such as *Akkermansia* (Zhu et al., 2021). *Pueraria lobata* polysaccharide (PPL), a root extract from *Pueraria montana var*. *lobata* (Willd.) Maesen and S.M.Almeida ex Sanjappa and Predeep, improves UC by modulating the PI3K/Akt signaling pathway. It reduces the levels of pro-inflammatory factors (e.g., IL-6, IL-1β, TNF-α) in the intestine, thereby attenuating the inflammatory response. The administration of PPL improves the structure of the gut microbiota of UC mice by decreasing the relative abundance of Gram-negative bacteria. According to Spearman correlation analysis, PPL may affect metabolite production by modulating the intestinal microbiota and improving the entire intestinal environment (Zhang Z. et al., 2023). Rhein is an active metabolite obtained from the *Rheum palmatum* L. Studies have shown that Rhein can protect the intestinal mucosal barrier, reduce oxidative stress, and have anti-inflammatory effects by inhibiting the PI3K/Akt/mTOR signaling pathway, reducing the relative abundance of pathogenic bacteria *Enterobacteriaceae* and *Turicibacter*, and increasing the relative abundance of the probiotics *Unspecified-S24-7* and *Rikenellaceae* (Dong et al., 2022). Quzhou Aurantii Fructus Flavonoids (Zhishi) is a traditional Chinese herbal medicine, and its flavonoid extract Quzhou *Citrus × aurantium* L., whose main active metabolites are naringenin and hesperidin and more, has a protective effect on DSS-induced UC model in rats. It restores the relative abundance of probiotics *Lachnospiraceae_NK4A136_group* and *Alloprevotella*, reduces the abundance of pathogenic *Escherichia-Shigella* and *Parabacteroides*, and restores immune homeostasis by regulating the PI3K/Akt signaling pathway (Wang et al., 2025). Ginseng-derived nanofibers (GNFs) extracted from *Panax ginseng* C.A.Mey. (Renshen) modulate the mechanism of inhibition of the TLR4-mediated PI3K/Akt/NF-κB signaling pathway to reduce inflammation. GNFs significantly modulate the composition of the gut microbiota by enriching beneficial genera such as *Muribaculum*, *Lachnoclostridium*, and *NK4A214_group* while decreasing pathogenic genera including *Parasutterella* and *Alloprevotella* (Liu et al., 2025b). Isovitexin, a key anti-inflammatory metabolite isolated from *Dendrobium officinale Kimura and Migo* (Tiepi Shihu), significantly modulates the gut microbiota composition in UC mice. It increases the relative abundance of beneficial taxa, including butyric acid-producing *Odoribacter* and CAG-485, while reducing pathogenic *Firmicutes-D*, which negatively correlates with valeric acid levels. This microbiota remodeling leads to elevated concentrations of butyric and valeric acids and enhances the intestinal metabolic capacity for SCFAs. Mechanistically, isovitexin has been shown to alleviate UC symptoms via inhibition of the PI3K/Akt signaling pathway, restoration of the intestinal barrier, and regulation of microbial homeostasis (Dai et al., 2025).

In summary, botanical drugs and their metabolites or extracts have shown multidimensional therapeutic potential for UC through regulation of the PI3K/Akt pathway. For example, *Bletilla striata* (Thunb.) Rchb.f. and BDMC exhibit anti-inflammatory effects. DHE and PEO exhibit anti-inflammatory, anti-oxidative stress, and gut microbiota-regulating properties, while BI, PPL, Quzhou Aurantii Fructus Flavonoids, and isovitexin exhibit anti-inflammatory effects, repair the intestinal barrier, and regulate the gut microbiota. However, the available evidence is predominantly derived from animal models and cell-based studies, with notable limitations in dose determination, compound stability, and methodological rigor that require further refinement.

## 5 Potential of TCM formulas in UC treatment

TCM formulas are known for their multilevel therapeutic approach to UC. They target multiple targets, cut across multiple signaling pathways, and exhibit diverse therapeutic effects. A prominent feature of Chinese medicines is their extremely low toxicity and side effects. In addition, the ability of Chinese medicines to enhance the body’s immune defenses and to significantly reduce the risk of drug resistance has made them an area of increasing interest in research in recent years. Experiments have demonstrated that TCM formulas have significant efficacy in regulating the balance of pro-inflammatory and anti-inflammatory factors, reducing the level of oxidative stress, regulating autophagy and apoptosis, repairing the intestinal mucosal barrier, and regulating the balance of intestinal microorganisms, which are achieved by modulating the PI3K/Akt signaling pathway (Supplementary Table 2).

### 5.1 Modulation of the inflammatory response

Qingzi Zhitong decoction (QZZTD) is a formula of five Chinese medicines that targets and regulates the PI3K-Akt signaling pathway in the treatment of UC. Studies have shown that key metabolites in QZZTD, such as quercetin, luteolin, mandarin, and beta-sitosterol, bind to Akt1 protein and inhibit the activation of the PI3K-Akt pathway. This, in turn, attenuates the action of the pro-inflammatory factor TNF-α and achieves an anti-inflammatory effect (Shou et al., 2022). Gegen Qinlian Decoction (GQD), a classical Chinese herbal formula, has good therapeutic effects on UC. In the DSS-induced UC animal model, GQD significantly reduced the expression of EGFR, PI3K, and p-Akt proteins, thereby inhibiting the overactivation of the PI3K-Akt signaling pathway. Meanwhile, GQD also inhibited the expression of pro-inflammatory cytokines, such as TNF-α, IL-1β, and IL-6, and attenuated inflammation and tissue damage in the colon (Liu et al., 2021). Fructus mume pills (FMPs) have been recommended for the treatment of ascaridiasis and chronic diarrhea in China since 200 AD. FMPs reduce intestinal inflammation by regulating the secretion of TNF-α, IL-6, IL-8, and IL-10 through the VEGF-PI3K/Akt-eNOS pathway. In addition, FMPs promote intestinal vascular repair and enhance blood flow through this pathway. This is manifested by VEGF activating the PI3K/Akt pathway, which further activates eNOS, increasing nitric oxide synthesis, dilating blood vessels, improving blood circulation, and promoting the repair and healing of damaged intestines (Xu et al., 2022).

### 5.2 Reduction of oxidative stress

Kuijieyuan Decoction (KD) is a combination of nine botanical drugs with anti-inflammatory, antioxidant, and antibacterial properties. Studies have shown a strong correlation between the levels of PF and BI occupying more proportions and oxidative stress biomarkers in KD. KD significantly reduced the release of pro-inflammatory cytokines TNF-α, IL-1β, and IL-6, while up-regulating the expression of anti-inflammatory cytokine IL-10 by inhibiting the over-activation of PI3K/Akt/NF-κB signaling pathway. In addition, KD enhances the activity of antioxidant enzymes, including SOD, CAT, and GPx, and reduces the level of MDA, a marker of oxidative stress. With the above mechanisms of action, KD is able to effectively attenuate inflammation and regulate the balance of oxidative stress, thus realizing the effective treatment of UC (Liu et al., 2020).

### 5.3 Regulation of autophagy and apoptosis

Renshen Baidu Powder (RBP) is a classic TCM formula used in the treatment of UC. Studies have demonstrated that RBP regulates the PI3K/Akt/NF-κB signaling pathway to suppress the release of pro-inflammatory cytokines such as TNF-α, IL-1β, and IL-6. In addition, RBP alleviates excessive apoptosis of colonic epithelial cells by downregulating the pro-apoptotic protein Bax and upregulating the anti-apoptotic protein Bcl-2, thereby maintaining intestinal homeostasis and restoring mucosal barrier function (Zhang P. et al., 2022). Compound Sophorae Decoction (Fufang Kushen Tang, CSD) is a classic formula in TCM. According to TCM principles, CSD is believed to have multiple functions, such as clearing heat, removing dampness, cooling the blood, and stopping diarrhea. Transmission electron microscopy (TEM) analysis of DSS-induced autophagic vacuoles in mice was almost non-existent. CSD enhanced the expression of autophagy-related proteins Atg5, Atg7, and Beclin1 by inhibiting the PI3K-Akt/mTOR pathway, and the protein levels of LC3II were significantly increased, while the protein level of p62 was significantly decreased. In addition, the expression of BCL2 was reduced, apoptosis was alleviated, and these effects helped to improve intestinal mucosal damage in UC (Liu et al., 2025a).

### 5.4 Repair of the intestinal barrier

Pulsatilla Decoction (PD) is derived from Zhang Zhongjing’s “Treatise on Typhoid Fever” and consists of four important botanical drugs. PD reduced Beclin1 and LC3 expression levels and significantly inhibited autophagy. It regulated the expression of intestinal epithelial tight junction proteins, increased the levels of Occludin and ZO-1, decreased the expression of Claudin-2, and improved intestinal barrier function. Moreover, PD significantly reduced the levels of IL-13 in serum and MPO in colonic tissues and repaired intestinal mucosal inflammation (Wang et al., 2022b). Sishen Pill (Sishen Wan, SSP) repairs the colon mucosal barrier by inhibiting the PI3K/Akt pathway, which in turn inhibits the downstream Rho/ROCK pathway. This increases the levels of tight junction proteins (Claudin-5 and JAM1), gap junction proteins (CX43), and adherens junction proteins (VE-cadherin) and reduces the levels of IL-1β and TNF-α (Zhang et al., 2021). Qingchang Wenzhong Decoction (QCWZD) consists of 8 commonly used Chinese medicines, which significantly upregulate the colon serum MSP/RON signaling pathway and repair macrophage inflammatory response and epithelial cell damage. Then it inhibits the PI3K/Akt signaling pathway, upregulates the expression of the key tight junction proteins Occludin and ZO-1, and downregulated the expression of Claudin-2, thus inhibiting intestinal inflammation, improving intestinal barrier function and ultimately repairing intestinal tissues (Mao et al., 2017).

### 5.5 Regulation of gut microbiota balance

Huangqin Decoction (HQD) significantly alleviated UC through multiple mechanisms. HQD inhibited the activity of the NF-κB signaling pathway by inhibiting the Ras-PI3K-Akt-HIF-1α signaling pathway, which in turn inhibited the M1 macrophage phenotypic markers iNOS and CXCL10 and the pro-inflammatory cytokines IL-1β and IL-6, while up-regulating the M2 macrophage phenotypic markers MR2 and Trem2 with the anti-inflammatory cytokines IL-4 and IL- 10, significantly reduced inflammation in the colon. HQD also exerted its anti-inflammatory effects by modulating the composition of the intestinal microbiota, which improved the intestinal microenvironment by increasing the diversity and relative abundance of beneficial bacteria and reducing the abundance of pathogenic bacteria (Li et al., 2020). KD ameliorated UC-induced intestinal barrier damage by inhibiting the overactivation of the PI3K/Akt/NF-κB signaling pathway, attenuating the inflammatory response, including restoration of the intestinal villi and mitochondrial structure, and modulating the gut microbiota (increasing *Alloprevotella*, *Treponema*, *Prevotella* and *Prevotellaceae*, and decreasing *Escherichia_Shigella* and *Desulfovibrio*) to optimize further the intestinal microenvironment (Liu et al., 2020). The TCM formula Zuojin Pill (ZJP) is a Chinese herbal prescription composed of *Coptis chinensis* Franch. and *Tetradium ruticarpum* (A.Juss.) T.G.Hartley in the ratio of 6:1. ZJP significantly regulated the composition of the intestinal microbiota and the number of CD4^+^CD25+Foxp3+ Treg cells, and enhanced the expression of PD-1 and PD-L1, as well as the immunosuppressive function of Treg cells. ZJP promoted the abundance of *Desulfovibrionaceae* and *Verrucomicrobiaceae*, selectively increasing some microbiota positively associated with PD-1^+^/PD-L1^+^ Treg cells, such as *Actinobacteria*, *Betaproteobacteria* and *Sphingobacteriia*. These results suggest that ZJP alleviates DSS-induced colitis by modulating the interaction between gut microbiota and Treg cell numbers through PI3K/Akt signaling lines (Zhou et al., 2020).

Overall, TCM formulas, with their multi-component, multi-target, and multi-pathway synergistic actions, exhibit notable advantages in intervening in UC through the PI3K/Akt pathway at multiple levels. However, the current evidence is almost exclusively derived from animal models and *in vitro* studies, and systematic clinical trials are still lacking, leaving their efficacy and safety unvalidated. Future research should prioritize large-scale, multicenter randomized controlled trials to facilitate clinical translation.

## 6 Conclusion and outlook

Chinese medicines take advantage of their multi-component synergy to target the PI3K/Akt signaling pathway. This dual-action approach not only regulates the balance between pro-inflammatory and anti-inflammatory cytokines, but also enhances the activity of antioxidant enzymes (SOD and GSH). It also repairs the intestinal mechanical barriers (Occludin and ZO-1) and the intestinal microbiota, showing the potential for multidimensional regulation of the “inflammation-oxidation-barrier-microbiota” axis. In addition, the herbal formula exerts its therapeutic effects through a multi-target mechanism. TCM formulas such as SSP inhibit the PI3K/Akt pathway. Then the downstream Rho/ROCK pathway, and HQD inhibits the activity of the NF-κB signaling pathway by inhibiting the Ras-PI3K-Akt-HIF-1α signaling pathway, achieving synergistic and increase the effect, highlighting the unique value of the holistic treatment concept of Chinese medicine.

Utilizing biomarkers to assess TCM quality is critical to enhancing its existing quality control standards and achieving standardization (Lu et al., 2022; Wangchuk et al., 2024). The unique advantage of TCM in treating UC through the PI3K/Akt signaling pathway has been initially validated in modern studies. The quantitative detection of the expression of proteins related to the conduction of the PI3K/Akt pathway, such as p-Akt, p-PI3K, Bcl-2, and Caspase-3, can provide objective and quantifiable biological indicators for the effect of TCM intervention. The study of TCM treatment of UC through the PI3K/Akt pathway enriches the modern therapeutic mechanism of TCM in chronic inflammatory diseases, provides a scientific basis for its clinical application, and practically advances the process of TCM standardization.

Although many natural products can alleviate UC through the PI3K/Akt pathway, their clinical application is often limited by low bioavailability, rapid metabolism, and poor targeting. To overcome these problems, researchers have developed various novel drug delivery systems in recent years, including pH-responsive colon-targeted granules, protein-based nanoparticles, and targeted-responsive liposomes, to improve the stability and targeted delivery of natural products *in vivo* (Gao et al., 2025). For example, pH-responsive particles of BI prepared with Eudragit S100 enteric coating polymer enable targeted release from the colon, significantly inhibiting the inflammatory response while reducing the required dose (Huang et al., 2024). RSV is delivered via beta-lactoglobulin nanospheres, which improves its stability and solubility and enhances its anti-inflammatory effects (Pujara et al., 2021). OMT loaded nitric oxide-releasing liposomes effectively alleviate the inflammatory response and promote the repair of the intestinal mucosal barrier (Tang et al., 2020). To address the challenge of metabolite delivery of natural products, the researchers developed a GGQL nano-preparation based on a hyaluronic acid-chitosan dual-responsive copolymer. The system co-delivers four active metabolites, BBR, Puerarin, BI, and Glycyrrhizin, to trigger the precise release of the drugs by taking advantage of the pathological microenvironment of high ROS and low pH at the site of colonic inflammation. This mechanism significantly increases the drug concentration at the lesion site, prolongs the local retention time, and relieves colitis by activating macrophage M2 polarization (Li et al., 2025). These technological tools have enhanced the biological effects of natural products in UC treatment at different levels, providing theoretical and practical support for their modern development and standardized evaluation.

At present, several limitations remain in current research. In TCM interventions in animals and cells, there is still a general problem of unclear basis for dose setting, and the subsequent studies need to standardize the dose selection and reporting criteria further. Second, many studies suffer from methodological shortcomings, such as inadequate control groups, short intervention periods, and a lack of systematic evaluation of long-term efficacy and mechanisms, which reduce the reproducibility and reliability of the findings. Third, most mechanistic studies are based on animal models, while pharmacodynamic and metabolic differences in humans remain unclear, and the absence of clinical validation limits translational value. Therefore, large-scale, multicenter randomized controlled trials are urgently needed to confirm efficacy and safety. Finally, the *in vivo* stability and bioavailability of active compounds remain low, leading to insufficient therapeutic durability and targeting. Addressing these issues will require establishing systematic dosage guidelines and quality control standards, together with the development of novel drug delivery systems. Future studies should also strengthen experimental design, extend observation periods, and integrate omics technologies and systems pharmacology to further elucidate the synergistic mechanisms of multi-component and multi-target actions. Through these improvements, the therapeutic potential of TCM in UC will become more clearly defined and more effectively translated from experimental studies into clinical practice.
