# Supplementary material for: Targeting the PI3K/Akt pathway for the treatment of ulcerative colitis: integrative regulatory features of traditional Chinese medicine
Source: Front Pharmacol. 2025 Oct 9;16:1620138. doi: 10.3389/fphar.2025.1620138 (PMC12546141; doi:10.3389/fphar.2025.1620138)
Supplement: Supplementary file 1 [file DataSheet1.pdf]

Supplementary Material

1 Supplementary Data

1.1 Supplementary Tables

Supplementary Table 1. Active metabolites of TCM interventions targeting UC

Name	Source	Study type	Animal /cell model	Dosage and duration	Optimal Dose	Control group	Mechanisms	Reported targets	Result	Reference
Citrus unshiu peel water extract	*Citrus reticulata* Blanco [Rutaceae; Citri Reticulatae Pericarpium] (Chen pi)	In vivo	DSS-induced UC in Balb/c mice	100 and 200 mg/kg*9 d	200 mg/kg	Sulfasalazine	PI3K/Akt	iNOS、Cox-2、TNF-α、IL-6、IL-1β↓; ROS、NOX2、MDA、NADPH↓; Bax、caspase-3↓, Bcl-2 and survivin↑	Modulated inflammatory response; Alleviated oxidative stress; Regulated apoptosis	(Lee et al., 2022)
Resveratrol	*Smilax glabra* Roxb. [Smilacaceae; Smilacis Glabrae Rhizoma] (Tufuling)	In vivo	DSS-induced UC in BALB/c mice	100 mg/kg*7 d	-	Sulfasalazine	PI3K/Akt/VEGFA	IL-6、IL-8、IL-1β、TNF-α↓, IL-10↑; *Clostridium*、*Roseburia*、*Akkermansia*↑	Modulated inflammatory response; Restored gut microbiota equilibrium	(Zhu et al., 2021)
Herba Origani Extract	*Origanum vulgare* L. [Lamiaceae;	In vivo	DSS-induced UC	0.125, 0.25, 0.5 and 1g/kg*10 d	-	Sulfasalazine	PI Pathway	IL-1β、TNF-α↓; Claudin-1、Occludin↑;	Modulated inflammatory response;	(Yu et al.,

Pulvis	Herba Origani] (Niu zhi)		in C57BL/6 mice					*Bacteroidota*，*Prevotellaceae UCG-001*、*Ruminococcaceae*↑，*Escherichia-Shigella*、*Desulfovibrio*、*Firmicutes/Bacteroidetes*↓; SCFAs、Deoxycholic acid、Hyodeoxycholic acid↑	Restored gut microbiota equilibrium; Repaired the intestinal barrier; Regulated bile acid and SCFA metabolism	2023)
Paeoniflorin	*Paeonia lactiflora* Pall. [Paeoniaceae; Paeoniae Radix Alba] (Baishao)	In vivo	DSS-induced UC in C57BL/6 mice	25, 50 and 100 mg/kg*7 d	100 mg/kg	Mesalazine	PI3K-Akt-mTOR	TNF-α、IL-1β↓; Lgr5、Sox9 and Ascl2↑; Ki67、ChgA、Muc2、Lyz、Villin↑; PARP1/PARP1、Bax↓; Bcl-2↑	Modulated inflammatory response; Regulated apoptosis; Promoted renewal and differentiation of intestinal stem cells to improve growth and repaired of colonic-like organs	(Ma et al., 2023)
		In vitro	LPS-injured IEC-6 cells	25, 50 and 100 μM*48 h	-	-				
		In vitro	TNF-α-induced colon organoids	25, 50, 100, and 200 μM*72 h	-	MK-2206、Rapamycin				
-	*Bletilla striata*	In vivo	DSS-induced	1, 2 and 4 g/kg*7 d	4 g/kg	Mesalazine	EGFR/PI3K/Akt	TNF-α、IL-6↓	Modulated inflammatory	(Gong

	(Thunb.) Rchb.f. [Orchidaceae; Bletillae Rhizoma] (Baiji)		UC in C57BL/6 mice						response	et al., 2023)
Bisdemethoxycurcumin	*Curcuma longa* L. [Zingiberaceae; Curcumae Longae Rhizoma] (Jianghuang)	In vitro	LPS-stimulated RAW264.7 cells	2.5 μM*25 h	2.5 μM	-	PI3K/Akt	IL-6、IL-1β、TNF-a、MCP-1↓	Modulated inflammatory response	(Wu et al., 2023)
Mogroside V	*Siraitia grosvenorii* (Swingle) A.M.Lu & Z.Y.Zhang [Cucurbitaceae; Siraitiae Fructus] (Luohanguo)	In vivo	DSS-induced in ICR mice	100 mg/kg/*14 d	-	Sulfasalazine	AMPK-PI3K/Akt/mTOR	IL-1β、TNF-α、IL-6、COX-2、AP-1/HO-1、iNOS↓	Modulated inflammatory response	(Zhou et al., 2021)
		In vitro	LPS-stimulated RAW264.7 cells	25, 50 and 100 μg/mL*16 h	-	Dexamethasone				
Dehydroevodiamine	*Tetradium ruticarpum* (A.Juss.) T.G.Hartley [Rutaceae; Evodiae Fructus] (Wuzhuyu)	In vivo	DSS-induced UC in SD mice	20 and 40 mg/kg*7 d	-	5-ASA	PI3K/Akt/NF-κB	IL-1β、IL-6、TNF-α↓; MDA↓, HO-1、SOD↑; ZO-1、occludin↑; Bax and Caspase-3↓; *Allobaculum*、	Modulated inflammatory respons; Repaired the intestinal barrier; Alleviated oxidative stress; Restored gut	(Ma et al., 2024)

								Clostridium、*Escherichia*、*Enterococcusand*、*Barnesiella*↓, *Lactobacillus*、*Bifidobacteriuma ndSMB53*↑	microbiota equilibrium;	
Codonopsis pilosula aqueous extract	*Codonopsis pilosula* (Franch.) Nannf. [Campanulaceae; Codonopsis Radix] (Dangshen)	In vivo	TNBS induced UC in SD rats	4.5, 9 and 18 g/kg*7 d	-	Sulfasalazine	PI3K/Akt	IL-1β、IL-6、IL-8、IL-17、PCT、GRP↓; GSH、SOD↑, MDA、MPO↓; ATPase↑	Modulated inflammatory response ; Alleviated oxidative stress;	(Li et al., 2024)
Patchouli Essential Oil	*Pogostemon cablin* (Blanco) Benth. [Lamiaceae; Pogostemonis Herba] (Guanghuoxiang)	In vivo	DSS-induced UC in ICR mice	25, 50 and 100 mg/kg*11 d	100 mg/kg	-	PI3K/Akt	TNF-α、IL-1β↓; GSH↑, MPO↓; *Bacteroidetes*、*Verrucomicrobia*、*S24-7*、*Lactobacillus*、*Prevotella and Akkermansia*↑, *Firmicutes*、*Actinobacteria*、*Staphylococcaceae*、*Corynebacteriaceae*、*Staphylococcus*、	Modulated inflammatory response; Alleviated oxidative stress; Restored gut microbiota equilibrium	(Huang et al., 2025)

Sanguisorba officinalis ethyl acetate extract	*Sanguisorba officinalis* L. [Rosaceae; Sanguisorbae Radix] (Diyu)	In vivo	DSS-induced UC in ICR mice	5, 10 and50 mg/kg*7 d	50 mg/kg	5-ASA	PI3K-Akt/NF-κB/STAT3	*Lactobacillus*↓ TNF-α、IL-1β、IL-6、iNOS↓; fibroblasts/macrophages↓, granulocytes、T cells↑; mucin glycoprotein↑	Modulated inflammatory response; Regulated macrophages; Repaired the intestinal barrier	(Li et al., 2023)
		In vitro	LPS-stimulated RAW264.7 cell	-	-	-				
Platycodin D	*Platycodon grandiflorum* (Jacq.) A.DC. [Campanulaceae; Platycodonis Radix] (Jiegeng)	In vivo	DSS-induced UC in C57BL/J mice	10 mg/kg*14 d	-	-	PI3K-Akt	TNF-α、IL-6、IL-1β↓, IL-10↑; iNOS、CD86↓, Arg1、CD206↑; TJP1、OCLN↑	Modulated inflammatory response; Regulated macrophage polarization; Repaired the intestinal barrier	(Guo et al., 2021)
		In vitro	LPS-induced in RAW 264.7 cells	2.5 and 5 μM*18 h	-	-				
Oxymatrine	*Sophora flavescens* Aiton [Fabaceae; Sophorae Flavescentis Radix] (Kushen)	In vivo	DSS-induced UC in BALB/c mice	25, 50, 100 mg/kg*7 d	50 mg/kg	LY294002	PI3K/Akt	TNF-α、IL-6、IL-1β、Th1/Th17↓、IL-10↑; Caspase3、Caspase9、Bad↑, Bcl-2↓	Modulated inflammatory response; Regulated cell apoptosis	(Chen et al., 2017)

Baicalin	*Scutellaria baicalensis* Georgi [Lamiaceae; Scutellariae Radix] (Huangqin)	In vivo	TNBS-induced UC in SD rats	100 mg/kg*14 d	-	LY294002、IGF-1	PI3K/Akt	TNF-α、IL-6、IL-1β↓，IL-10↑; HT-29、caspase-3、caspase-9、Bax、FasL↓, Bcl-2↑; β-catenin、ZO-1↑	Modulated inflammatory response; Inhibits of apoptosis in intestinal epithelial cells; Repaired the intestinal barrier	(Wang et al., 2022a)
		In vitro	LPS-induced HT-29 cells	-	-	LY294002、IGF-1				
-	*Cimicifuga heracleifolia* Kom. [Ranunculaceae; Cimicifugae Rhizoma] (Shengma)	In vivo	DSS-induced UC in C57BL/6 mice	0.17, 0.5 and 1.5 g/kg*7 d	1.5 g/kg	Sulfasalazine	PI3K/Akt/NF-κB	IL-6、IL-1β、TNF-a、iNOS、COX-2↓; Claudin-1、Occludin、ZO-1、Mucin-2↑	Modulated inflammatory response;, 。Repaired the intestinal barrier	(Wu et al., 2025)
Berberine	*Coptis chinensis* Franch. [Ranunculaceae; Coptidis Rhizoma] (Huanglian)	In vivo	DSS-induced UC in C57BL/6 mice	25, 50 and 100 mg/kg*7 d	100 mg/kg	Sulfasalazine	PI3K/Akt/mTOR	TNF-α、IL-6、IL-1β↓; DAO、D-LA↓	Modulated inflammatory response	(Meng et al., 2024)
		In vitro	LPS-induced RAW264.7 cells	3.125, 6.25, and 12.5 μM*24h	-	Small interfering RNA				

Astragaloside IV	*Astragalus membranaceus* Bunge [Fabaceae; Astragali Radix] (Huangqi)	In vivo	DSS-induced UC in C57BL/6 mice	100 and 150 mg/kg*10 d	-	5-ASA	PI3K/Akt	IL-6、IL-1β、TNF-α↓; ZO-1、Occludin、Claudin-5、Claudin-7、Villin↑; Claudin-2↓; *Alloprevotella*、*ruminococcaceae_UCG-014*、*Akkermansia*↑	Modulated inflammatory response;, Repaired the intestinal barrier; Restored gut microbiota equilibrium	(Zhang et al., 2024)
		In vitro	LPS-stimulated Caco-2 cells	150 μM*24h	-	LY294002				
Aloin A	*Aloe vera* (L.) Burm.f. [Asphodelaceae; Aloe] (Luhui)	In vivo	DSS-induced UC in SD rats	18 and 72 mg/kg*10 d	-	5-ASA	PI3K/Akt	IL-6、IL-1β、NO、TNF-α↓, IL-10↑; MUC2、MUC5AC↑	Modulated inflammatory response; Repaired the intestinal barrier	(Shi et al., 2021)
		In vitro	LPS-stimulated LS174T cells	-	-	-				
Pueraria lobata polysaccharide	*Pueraria montana var. lobata* (Willd.) Maesen & S.M.Almeida ex Sanjappa & Predeep [Fabaceae; Puerariae Lobatae	In vivo	DSS-induced UC in C57BL/6 mice	50, 100 and 200mg/kg*7 d	-	5-ASA	PI3K/Akt	IL-6、IL-1β、TNF-a↓; Caco-2↓; ZO-1、occludin、claudin-1↑	Modulated inflammatory response; Repaired the intestinal barrier ; Restored gut microbiota equilibrium	(Zhang et al., 2023)
		In vitro	DSS-induced Caco-2 cells	0, 10, 20, and 40 μg/mL*24h	-	PI3K siRNA				

	Radix] (Gegen)									
Rhein	*Rheum palmatum* L. [Polygonaceae; Rhei Radix et Rhizoma] (Dahuang)	In vivo	DSS-induced UC in BALB/c mice	12.5, 25 and 50 mg/kg*7 d	50 mg/kg		PI3K/Akt/mTOR	TNF-α、IL-6、IL-1β↓; *Unspecified-S24-7*、*Rikenellaceae*↑, *Enterobacteriaceae*、*Turicibacter*↓	Modulated inflammatory response; Restored gut microbiota equilibrium	(Dong et al., 2022)
		In vitro	LPS-stimulated RAW 264.7 cells	10, 20 and 40 μM* 25 h	-	SC79				
Quzhou Aurantii Fructus Flavonoids	*Citrus* × *aurantium* L. [Rutaceae; Aurantii Fructus Immaturus] (Zhishi)	In vivo	DSS-induced UC in C57BL/6 mice	50, 100 and 200 mg/kg*11 d	-	5-ASA	PI3K/Akt	TNF-α, IL-6、IL-1β↓，IL-10↑;Claudin-1、Occludin、ZO-1、Claudin-1↑; *Lachnospiraceae_NK4A136_group*、*Alloprevotella*↓，*Escherichia-Shigella*、*Parabacteroides*↑; SCFAs、PE、PC↑	Modulated inflammatory response; Repaired the intestinal barrier; Restored gut microbiota equilibrium	(Wang et al., 2025)

Ginseng-derived nanocellulose	*Panax ginseng* C.A.Mey. [Araliaceae; Ginseng Radix et Rhizoma] (Renshen)	In vivo	DSS-induced UC in BALB/c mice	10, 25 and 50 mg/kg*7 d	-	-	PI3K/Akt-NF-κB	TNF-α、IL-6、IL-1β、IL-17↓;ZO-1、Occludin、MUC2↑; *Muribaculum*、*Lachnoclostridium*、*NK4A214_group*、*Desulfovibrio*↑, *Alloprevotell*、*Parasutterella*↓	Repaired the intestinal barrier; Restored gut microbiota equilibrium	(Liu et al., 2025b)
		In vitro	LPS-induced in RAW 264.7 cells	15.625-500μg/mL*24h	-	-				
		In vitro	Caco-2 cells and organoids	15.625, 31.25, 62.5, 125, 250, and 500μg/mL*24 h 62.5, 125, 250 μg/mL*24 h	-	-				
Isovitexin	*Dendrobium officinale* Kimura & Migo [Orchidaceae; Dendrobii Officinalis Caulis] (Tiepi Shihu)	In vivo	DSS-induced UC in C57BL/6 mice	50 and 100 mg/kg*7 d	-	-	PI3K/Akt	IL-1β、IL-6、TNF-α↓;ZO-1、Occludin↑;SCFAs↑; *Firmicutes/Bacteroidetes*、*Odoribacter*、*CAG-485*、*Parabacteroides*	Modulated inflammatory response; Repaired the intestinal barrier; Restored gut microbiota equilibrium	(Dai et al., 2025)

	In vitro	LPS-induced in RAW 264.7 cells	400 μg/mL*24 h	-	Indomethacin		、*Cryptobacteroides*、*Duncaniella*、*UBA3263*、*Limosilactobacillus*、*Aerococcus*↑;*Bacteroides*、*Prevotella*、*Bacteroides*、*Phocaeicola*、*Ruminococcus*↓	

Supplementary Table 2. TCM formulas targeting UC treatment

Form	Formula	Study type	Animal/cell model	Dosage and duration	Optimal Doses	control group	Mechanisms	Reported targets	result	Refe ce
Baphicacanthus cusia (Nees) Bremek. [Acanthaceae; Indigo Naturalis] (Qingdai) *Arnebia euchroma* (Royle ex Benth.) I.M.Johnst.	Qingzi Zhitong decoction	In vivo	TNBS/ethanol mixture induced UC in SD rats	8.6 g/kg*10d	-	-	PI3K/Akt	TNF-α、IL-6、IL-1β↓	Modulated inflammatory response	(Sh et a 202

[Boraginaceae; Arnebiae Radix] (Zicao) *Bletilla striata* (Thunb.) Rchb.f. [Orchidaceae; Bletillae Rhizoma] (Baiji) *Corydalis yanhusuo* W.T.Wang [Papaveraceae; Corydalis Rhizoma] (Yuanhu) *Reynoutria japonica* Houtt. [Polygonaceae; Polygoni Cuspidati Rhizoma et Radix] (Huzhang)										
Pueraria montana var. lobata (Willd.) Maesen & S.M.Almeida ex Sanjappa & Predeep [Fabaceae;	Gegen Qinlian Decoction	In vivo	DSS-induced UC in C57BL/6 mice	0.68 g/kg*7d	-	Sulfasalazine	EGFR/PI3K/AKT	TNF-α、IL-1β、IL-6↓	Modulated inflammatory response	(Liu al 202

Puerariae Lobatae Radix] (Gegen) *Scutellaria baicalensis* Georgi [Lamiaceae; Scutellariae Radix] (Huangqin) *Coptis chinensis* Franch. [Ranunculaceae; Coptidis Rhizoma] (Huanglian) *Glycyrrhiza uralensis* Fisch. ex DC. [Fabaceae; Glycyrrhizae Radix et Rhizoma] (Gancao)										
Prunus mume (Siebold) Siebold & Zucc. [Rosaceae; Mume Fructus] (Wumei) *Zingiber officinale*	Fructus mume pills (Wumei Wan)	In vivo	AA-induced UC in SD rats	450 and 900 mg/kg*7d	900 mg/kg	-	VEGF-PI3K/Akt-eNOS	TNF-α、IL-6、IL-8↓，IL-10↑; MPO、MMP9、CXCR1↓	Modulated inflammatory response; Regulated microvascular permeability	(Xu[illegible] al[illegible] 202[illegible]

Roscoe [Zingiberaceae; Zingiberis Rhizoma] (Ganjiang) *Coptis chinensis* Franch. [Ranunculaceae; Coptidis Rhizoma] (Huanglian) *Asarum sieboldii* Miq.Herba [Aristolochiaceae; Asari Radix et Rhizoma] (Xixin) *Angelica sinensis* (Oliv.) Diels [Apiaceae; Angelicae Sinensis Radix] (Danggui) *Aconitum carmichaelii* Debeaux [Ranunculaceae; Aconiti Lateralis Radix Praeparata]

(Fuzi) *Zanthoxylum bungeanum* Maxim. [Rutaceae; Zanthoxyli Pericarpium] (Huajiao) *Cinnamomum cassia* (L.) J.Presl [Lauraceae; Cinnamomi Ramulus] (Guizhi) *Codonopsis pilosula* (Franch.) Nannf. [Campanulaceae; Codonopsis Radix] (Dangsheng) *Phellodendron chinense* Schneid. [Rutaceae; Phellodendri Cortex] (Huangbai)										
Astragalus membranaceus Bunge [Fabaceae;	Kuijieyuan Decoction	In vivo	DSS-induced UC in SD rats	1, 2 and 10 ml/kg*15d	10 ml/kg	sulfasalazine	PI3K/AKT/NF-kB	TNFα、IL-1、IL-6↓，IL-10↑; SOD、GPx、	Modulated inflammatory response; Alleviated	(Liu al 202

Astragali Radix] (Huangqi)<br>*Hedyotis diffusa* Willd. [Rubiaceae; Hedyotis Diffusae Herba] (Baihua Sheshecao)<br>*Cirsium undulatum* (Nutt.) Spreng [Asteraceae; wavyleaf thistle] (Boyeji)<br>*Cirsium arvense var. arvense* [Asteraceae; Cirsii Herba] (Xiaoji)<br>*Pulsatilla chinensis* (Bunge) Regel [Ranunculaceae; Pulsatillae Radix] (Baitouweng)<br>*Prunella vulgaris* L. [Lamiaceae; Prunellae	CAT↑，MDA↓; intestinal villi、mitochondrial ridges↑; Alloprevotella, Treponema, Prevotellaceae, and Prevotella↑，Escherichia_Shigella and Desulfovibrio↓	oxidative stress; Repaired the intestinal barrier; Restored gut microbiota equilibrium

Spica] (Xiakucao)
Coptis chinensis Franch. [Ranunculaceae; Coptidis Rhizoma] (Huanglian)
Polygonum cuspidatum Siebold & Zucc. [Polygonaceae; Polygoni Cuspidati Rhizoma et Radix] (Huzhang)
Atractylodes lancea (Thunb.) DC. [Asteraceae; Atractylodis Rhizoma] (Cangzhu)
Glycyrrhiza uralensis Fisch. ex DC. [Fabaceae; Glycyrrhizae Radix et Rhizoma] (Gancao)

Pulsatilla chinensis (Bunge) Regel [Ranunculaceae; Pulsatillae Radix] (Baitouweng) *Fraxinus chinensis subsp. rhynchophylla* (Hance) A.E.Murray [Oleaceae; Fraxini Cortex] (Qinpi) *Phellodendron chinense* C.K.Schneid. [Rutaceae; Phellodendri Cortex] (Huangbai) *Coptis chinensis* Franch. [Ranunculaceae; Coptidis Rhizoma] (Huanglian)	Pulsatilla Decoction (Baitouweng Tang)	In vivo	Oxazolone induced UC in C57BL/6 murine	20 mg/g*7d	-	5-ASA	PI3K-Akt-mTORC1	IL-13、MPO↓; Beclin1、LC3↓; Occludin、ZO-1↑, Claudin-2↓	Modulated inflammatory response; Regulated autophagy; Repaired the intestinal barrier	(Wa[illegible] et a[illegible] 202[illegible]
Bupleurum chinense DC. [Apiaceae; Bupleuri	Renshen Baidu Powder	In vivo	TNBS/ethanol mixture induced UC in SD	7.8 ,15.6 and 31.2 g/kg*14d	31.2 g/kg	sulfasalazine	PI3K/AKT/NF-κB	TNF-α、IL-1β、IL-6↓; Bax↑, Bcl-2↓	Modulated inflammatory response; Inhibited of	(Zha[illegible] et a[illegible] 202[illegible]

Radix] (Chaihu) *Ligusticum chuanxiong* Hort. [Apiaceae; Chuanxiong Rhizoma] (Chuanxiong) *Panax ginseng* C.A.Mey. [Araliaceae; Ginseng Radix et Rhizoma] (Renshen) *Kitagawia praeruptora* (Dunn) Pimenov [Apiaceae; Peucedani Radix] (Qianhu) *Glycyrrhiza uralensis* Fisch. ex DC. [Fabaceae; Glycyrrhizae Radix et Rhizoma] (Gancao) *Platycodon grandiflorus* (Jacq.) A.DC.	rats	apoptosis in intestinal epithelial cells; Repaired the intestinal barrier

[Campanulaceae; Platycodonis Radix] (Jiegeng)
Hansenia weberbaueriana (Fedde ex H.Wolff) Pimenov & Kljuykov [Apiaceae; Notopterygii Rhizoma et Radix] (Qianghuo)
Angelica pubescens Maxim. [Apiaceae; Angelicae Pubescentis Radix] (Duhuo)
Poria cocos (Schw.) Wolf [Polyporaceae; Poria] (Fuling)
Citrus × *aurantium* L. [Rutaceae; Aurantii Fructus Immaturus] (Zhike)

Mentha canadensis L. [Lamiaceae; Menthae Haplocalycis Herba] (Bohe) *Zingiber officinale* Roscoe [Zingiberaceae; Zingiberis Rhizoma Recens] (Shengjiang) *Sophora flavescens* Aiton [Fabaceae; Sophorae Flavescentis Radix] (Kushen) *Sanguisorba officinalis* L. [Rosaceae; Sanguisorbae Radix] (Diyu) *Strobilanthes cusia* (Nees) Kuntze [Acanthaceae; Indigo Naturalis] (Qingdai)	Compound Sophorae Decoction (Fufang Kushen Tang)	In vivo	DSS-induced UC in C57BL/6J mice	14.56 g/kg*7	-	bpV	PI3K-AKT/mTOR	IFN-γ、IL-1β、IL-6、TNF-α、TGF-β、IL-2↓, IL-10 、IL-4↑; Atg5、Atg7、Beclin1、LC3II↑, p62↓; Bcl-2↓, HIF-1α↑	Modulated inflammatory response; Alleviated oxidative stress; Regulated autophagy; Regulated cell apoptosis	(Liu [illegible] al [illegible] 202[illegible]

Bletilla striata (Thunb.) Rchb.f. [Orchidaceae; Bletillae Rhizoma] (Baiji) *Panax notoginseng* (Burkill) F.H. Chen (Sanqi) *Glycyrrhiza uralensis* Fisch. ex DC. [Fabaceae; Glycyrrhizae Radix et Rhizoma] (Gancao) *Tetradium ruticarpum* (A.Juss.) T.G.Hartley [Rutaceae; Evodiae Fructus] (Wuzhuyu) *Cullen corylifolium* (L.) Medik. [Fabaceae; Psoraleae Fructus] (Buguzhi)	Sishen Pill	In vivo	TNBS-induced UC in SD mice	5 g/kg*10d	-	5-ASA	PI3K/Akt	TNF-α、IL-1β↓;Claudin-5、JAM1、VE-cadherin、Connexin 43↑	Modulated inflammatory response; Repaired the intestinal barrier	(Zha[illegible] et a[illegible] 202[illegible]

Myristica fragrans Houtt. [Myristicaceae; Myristicae Semen] (Roudoukou) *Schisandra chinensis* (Turcz.) Baill. [Schisandraceae; Schisandrae Chinensis Fructu] (Wuweizi) *Coptis chinensis* Franch. [Ranunculaceae; Coptidis Rhizoma] (Huanglian) *Zingiber officinale* Roscoe [Zingiberaceae; Zingiberis Rhizoma Praeparatum] (Paojiang) *Sophora flavescens* Aiton [Fabaceae; Sophorae	Qingchang Wenzhong Decoction	In vivo	DSS-induced UC in SD mice	0.3, 0.6 and 1.2 g/kg*7d	0.6 g/kg	Mesalazine	PI3K/Akt	Occludin、ZO-1↑，claudin-2↓	Repaired the intestinal barrier	(Ma[illegible] al[illegible] 201[illegible]

Flavescentis Radix] (Kushen)
Baphicacanthus cusia (Nees) Bremek. [Acanthaceae; Indigo Naturalis] (Qingdai)
Sanguisorba officinalis L. [Rosaceae; Sanguisorbae Radix] (Diyutan)
Dolomiaea costus (Falc.) Kasana & A.K.Pandey [Asteraceae; Aucklandiae Radix] (Muxiang)
Panax notoginseng (Burkill) F.H.Chen [Araliaceae; Notoginseng Radix et Rhizoma] (Sanqi)
Glycyrrhiza

uralensis Fisch. ex DC. [Fabaceae; Glycyrrhizae Radix et Rhizoma] (Gancao) *Scutellaria baicalensis* Georgi [Lamiaceae; Scutellariae Radix] (Huangqin) *Glycyrrhiza uralensis* Fisch. ex DC. [Fabaceae; Glycyrrhizae Radix et Rhizoma] (Gancao) *Paeonia lactiflora* Pall. [Paeoniaceae; Paeoniae Radix Alba] (Baishao) *Ziziphus jujuba* Mill. [Rhamnaceae; Jujubae Fructus] (Dazao)	Huangqin Decoction	In vivo	DSS-induced UC in BALB/c mice	2.275, 4.55 and 9.1 g/kg*7d	9.1 g/kg	Mesalazine	Ras-PI3K-Akt-HIF-1a	iNOS、CXCL10、MR、Trem2、MPO;IL-1β and IL-6↓;Bacteroidetes and Lactobacillus↑, Proteobacteria、Ruminococcaceae、Lachnospiraceae↓	Modulated inflammatory response; Repaired the intestinal barrier; Restored gut microbiota equilibrium	(Li[illegible] al[illegible] 202[illegible]

Coptis chinensis Franch. [Ranunculaceae; Coptidis Rhizoma] (Huanglian) *Tetradium ruticarpum* (A.Juss.) T.G.Hartley [Rutaceae; Evodiae Fructus] (Wuzhuyu)	Zuojin Pill	In vivo	DSS-induced UC in BALB/c mice	1.5, 3 and 6g/kg*7d	3 g/kg	5-ASA	PI3K/Akt	IL-2 , IL-6, IL-17A、IL-4↓;PD-1、PD-L1↓;Regulatory T cells、Akkermansia and Actinobacteria↑	Modulated inflammatory response; Restored gut microbiota equilibrium	(Zh et a 202

Reference：

Chen, Q., Duan, X., Fan, H., Xu, M., Tang, Q., Zhang, L., et al. (2017). Oxymatrine protects against DSS-induced colitis via inhibiting the PI3K/AKT signaling pathway. *Int Immunopharmacol* 53, 149–157. doi: 10.1016/j.intimp.2017.10.025

Dai, J., Wang, W., He, F., Yu, X., Liu, Z., Wang, Y., et al. (2025). Discovery of anti-inflammatory molecules from Dendrobium officinale based on activity labelled molecular networking and its alleviation effect on ulcerative colitis. *Food Research International* 203, 115888. doi: 10.1016/j.foodres.2025.115888

Dong, L., Du, H., Zhang, M., Xu, H., Pu, X., Chen, Q., et al. (2022). Anti-inflammatory effect of Rhein on ulcerative colitis via inhibiting PI3K/Akt/mTOR signaling pathway and regulating gut microbiota. *Phytother Res* 36, 2081–2094. doi: 10.1002/ptr.7429

Gong, S., Lv, R., Fan, Y., Shi, Y., and Zhang, M. (2023). The potential mechanism of Bletilla striata in the treatment of ulcerative colitis determined through network pharmacology, molecular docking, and in vivo experimental verification. *Naunyn Schmiedebergs Arch Pharmacol* 396, 983–1000. doi: 10.1007/s00210-022-02370-9

Guo, R., Meng, Q., Wang, B., and Li, F. (2021). Anti-inflammatory effects of Platycodin D on dextran sulfate sodium (DSS) induced colitis and E. coli Lipopolysaccharide (LPS) induced inflammation. *International Immunopharmacology* 94, 107474. doi: 10.1016/j.intimp.2021.107474

Huang, Y., Zhao, P., Zhang, X., Fu, H., and Fu, C. (2025). Uncovering the pharmacological mechanisms of Patchouli essential oil for treating ulcerative colitis. *Journal of Ethnopharmacology* 336, 118737. doi: 10.1016/j.jep.2024.118737

Lee, S. H., Lee, J. A., Shin, M.-R., Park, H.-J., and Roh, S.-S. (2022). Citrus unshiu Peel Attenuates Dextran Sulfate Sodium-Induced Ulcerative Colitis in Mice due to Modulation of the PI3K/Akt Signaling Pathway and MAPK and NF-κB. *Evidence-Based Complementary and Alternative Medicine* 2022, 1–13. doi: 10.1155/2022/4041402

Li, C., Gong, L., Jiang, Y., Huo, X., Huang, L., Lei, H., et al. (2023). Sanguisorba officinalis ethyl acetate extract attenuates ulcerative colitis through inhibiting PI3K-AKT/NF-κB/ STAT3 pathway uncovered by single-cell RNA sequencing. *Phytomedicine* 120, 155052. doi: 10.1016/j.phymed.2023.155052

Li, F., Yang, Y., Ge, J., Wang, C., Chen, Z., Li, Q., et al. (2024). Multi-omics revealed the mechanisms of Codonopsis pilosula aqueous extract in improving UC through blocking abnormal activation of PI3K/Akt signaling pathway. *Journal of Ethnopharmacology* 319, 117220. doi: 10.1016/j.jep.2023.117220

Li, M., Luo, H., Wu, X., Liu, Y., Gan, Y., Xu, N., et al. (2020). Anti-Inflammatory Effects of Huangqin Decoction on Dextran Sulfate Sodium-Induced Ulcerative Colitis in Mice Through Regulation of the Gut Microbiota and Suppression of the Ras-PI3K-Akt-HIF-1α and NF-κB Pathways. *Front Pharmacol* 10, 1552. doi: 10.3389/fphar.2019.01552

Liu, B., Piao, X., Niu, W., Zhang, Q., Ma, C., Wu, T., et al. (2020). Kuijieyuan Decoction Improved Intestinal Barrier Injury of Ulcerative Colitis by Affecting TLR4-Dependent PI3K/AKT/NF-κB Oxidative and Inflammatory Signaling and Gut Microbiota. *Front Pharmacol* 11, 1036. doi: 10.3389/fphar.2020.01036

Liu, X., Fan, Y., Du, L., Mei, Z., and Fu, Y. (2021). In Silico and In Vivo Studies on the Mechanisms of Chinese Medicine Formula (Gegen Qinlian Decoction) in the Treatment of Ulcerative Colitis. *Front. Pharmacol.* 12, 665102. doi: 10.3389/fphar.2021.665102

Liu, Y., Deng, S., Sun, L., He, H., Zhou, Q., Fan, H., et al. (2025a). Compound sophorae decoction mitigates DSS-induced ulcerative colitis by activating autophagy through PI3K-AKT pathway: A integrative research combining network pharmacology and in vivo animal model validation. *Journal of Ethnopharmacology* 337, 118885. doi: 10.1016/j.jep.2024.118885

Liu, Y., Zhang, Z., Tao, S., Li, T., Wu, Y., Zhang, C., et al. (2025b). Ginseng-derived nanocellulose alleviates murine colitis through modulation of inflammation, epithelial barrier and gut microbiome. *Chemical Engineering Journal* 506, 160254. doi: 10.1016/j.cej.2025.160254

Ma, X., Hu, Q., Jiang, T., Chen, Y., Zhang, W., Gao, P., et al. (2024). Dehydroevodiamine Alleviates Ulcerative Colitis by Inhibiting the PI3K/AKT/NF-κB Signaling Pathway via Targeting AKT1 and Regulating Gut Microbes and Serum Metabolism. *Molecules* 29, 4031. doi: 10.3390/molecules29174031

Ma, Y., Lang, X., Yang, Q., Han, Y., Kang, X., Long, R., et al. (2023). Paeoniflorin promotes intestinal stem cell-mediated epithelial regeneration and repair via PI3K-AKT-mTOR signalling in ulcerative colitis. *Int Immunopharmacol* 119, 110247. doi: 10.1016/j.intimp.2023.110247

Mao, T., Li, J., Liu, L., Zhao, W., Liu, Y., Gao, K., et al. (2017). Qingchang Wenzhong Decoction Attenuates DSS-Induced Colitis in Rats by Reducing Inflammation and Improving Intestinal Barrier Function via Upregulating the MSP/RON Signalling Pathway. *Evid Based Complement Alternat Med* 2017, 4846876. doi: 10.1155/2017/4846876

Meng, G., Li, P., Du, X., Feng, X., and Qiu, F. (2024). Berberine alleviates ulcerative colitis by inhibiting inflammation through targeting IRGM1. *Phytomedicine* 133, 155909. doi: 10.1016/j.phymed.2024.155909

Shi, G., Jiang, H., Feng, J., Zheng, X., Zhang, D., Jiang, C., et al. (2021). Aloe vera mitigates dextran sulfate sodium-induced rat ulcerative colitis by potentiating colon mucus barrier. *Journal of Ethnopharmacology* 279, 114108. doi: 10.1016/j.jep.2021.114108

Shou, X., Wang, Y., Zhang, X., Zhang, Y., Yang, Y., Duan, C., et al. (2022). Network Pharmacology and Molecular Docking Analysis on Molecular Mechanism of Qingzi Zhitong Decoction in the Treatment of Ulcerative Colitis. *Front. Pharmacol.* 13, 727608. doi: 10.3389/fphar.2022.727608

Wang, H., Huang, W., Pan, X., Tian, M., Chen, J., Liu, X., et al. (2025). Quzhou Aurantii Fructus Flavonoids Ameliorate Inflammatory Responses, Intestinal Barrier Dysfunction in DSS-Induced Colitis by Modulating PI3K/AKT Signaling Pathway and Gut Microbiome. *JIR* 18, 1855–1874. doi: 10.2147/JIR.S500014

Wang, X., Xie, L., Long, J., Liu, K., Lu, J., Liang, Y., et al. (2022a). Therapeutic effect of baicalin on inflammatory bowel disease: A review. *Journal of Ethnopharmacology* 283, 114749. doi: 10.1016/j.jep.2021.114749

Wang, X., Xu, L., Wang, T., Xu, J., Fan, F., Zhang, Y., et al. (2022b). Pulsatilla decoction alleviates colitis by enhancing autophagy and regulating PI3K-Akt-mTORC1 signaling pathway. *Mol Med Rep* 25, 1–10. doi: 10.3892/mmr.2022.12624

Wu, H., Tu, S., Zhuo, Z., Jiang, R., Zeng, R., Yang, Q., et al. (2023). Investigating the Mechanisms of Bisdemethoxycurcumin in Ulcerative Colitis: Network Pharmacology and Experimental Verification. *Molecules* 28, 68. doi: 10.3390/molecules28010068

Wu, X.-Y., Dong, Q.-W., Zhang, Y.-B., Li, J.-X., Zhang, M.-Q., Zhang, D.-Q., et al. (2025). *Cimicifuga heracleifolia* kom. Attenuates ulcerative colitis through the PI3K/AKT/NF-κB signaling pathway. *Journal of Ethnopharmacology* 337, 118892. doi: 10.1016/j.jep.2024.118892

Xu, Z., Zhang, X., Lu, R., Zhang, D., Zou, T., Chen, M., et al. (2022). Mechanism of Fructus Mume Pills Underlying Their Protective Effects in Rats with Acetic Acid-Inducedulcerative Colitis via the Regulation of Inflammatory Cytokines and the VEGF-PI3K/Akt-eNOS Signaling Pathway. *Evid Based Complement Alternat Med* 2022, 4621131. doi: 10.1155/2022/4621131

Yu, Z., Li, D., and Sun, H. (2023). Herba Origani alleviated DSS-induced ulcerative colitis in mice through remolding gut microbiota to regulate bile acid and short-chain fatty acid metabolisms. *Biomedicine & Pharmacotherapy* 161, 114409. doi: 10.1016/j.biopha.2023.114409

Zhang, P., Zhang, X., Xiong, P., Zhong, C., Zhou, Z., Jia, B., et al. (2022). Renshen Baidu Powder Attenuated Intestinal Inflammation and Apoptosis in Ulcerative Colitis Rats through the Inhibition of PI3K/AKT/NF-κB Signaling Pathway. *Evidence-Based Complementary and Alternative Medicine* 2022, 5234025. doi: 10.1155/2022/5234025

Zhang, X., Zhang, F., Li, Y., Fan, N., Zhao, K., Zhang, A., et al. (2024). Blockade of PI3K/AKT signaling pathway by Astragaloside IV attenuates ulcerative colitis via improving the intestinal epithelial barrier. *Journal of Translational Medicine* 22, 406. doi: 10.1186/s12967-024-05168-w

Zhang, X.-Y., Zhao, H.-M., Liu, Y., Lu, X.-Y., Li, Y.-Z., Pan, Q.-H., et al. (2021). Sishen Pill Maintained Colonic Mucosal Barrier Integrity to Treat Ulcerative Colitis via Rho/ROCK Signaling Pathway. *Evidence-Based Complementary and Alternative Medicine* 2021, 5536679. doi: 10.1155/2021/5536679

Zhang, Z., Cui, Y., Ouyang, H., Zhu, W., Feng, Y., Yao, M., et al. (2023). Radix Pueraria lobata polysaccharide relieved DSS-induced ulcerative colitis through modulating PI3K signaling. *Journal of Functional Foods* 104, 105514. doi: 10.1016/j.jff.2023.105514

Zhou, B.-G., Liu, F.-C., Zhao, H.-M., Zhang, X.-Y., Wang, H.-Y., and Liu, D.-Y. (2020). Regulatory effect of Zuojin Pill on correlation with gut microbiota and Treg cells in DSS-induced colitis. *Journal of Ethnopharmacology* 262, 113211. doi: 10.1016/j.jep.2020.113211

Zhou, Y., Hu, Z., Ye, F., Guo, T., Luo, Y., Zhou, W., et al. (2021). Mogroside V exerts anti-inflammatory effect via MAPK-NF-κB/AP-1 and AMPK-PI3K/Akt/mTOR pathways in ulcerative colitis. *Journal of Functional Foods* 87, 104807. doi: 10.1016/j.jff.2021.104807

Zhu, F., Zheng, J., Xu, F., Xi, Y., Chen, J., and Xu, X. (2021). Resveratrol Alleviates Dextran Sulfate Sodium-Induced Acute Ulcerative Colitis in Mice by Mediating PI3K/Akt/VEGFA Pathway. *Frontiers in Pharmacology* 12, 693982. doi: 10.3389/fphar.2021.693982
